# Gyrification changes are related to cognitive strengths in autism

**DOI:** 10.1016/j.nicl.2018.04.036

**Published:** 2018-08-04

**Authors:** P. Duret, F. Samson, B. Pinsard, E.B. Barbeau, A. Boré, I. Soulières, L. Mottron

**Affiliations:** a Centre d'Excellence en Troubles Envahissants du Développement de l'Université de Montréal, (CETEDUM), Montréal, Canada; b Département de Neurosciences, Université de Montréal, Montréal, Canada; c École Normale Supérieure de Lyon, Lyon, France; d Brain Dynamics and Cognition, Lyon Neuroscience Research Center, INSERM U1028, CNRS UMR 5292, Lyon, France & University Lyon 1, F-69000 Lyon, France; e Unité de Neuroimagerie Fonctionnelle, Centre de Recherche de l'Institut Universitaire de Gériatrie de Montréal, Montréal, Canada; f Sorbonne Universités, UPMC Univ Paris 06, CNRS UMR 7371, INSERM UMR_S 1146, Laboratoire d'Imagerie Biomédicale, F-75013 Paris, France; g Département de Psychologie, Université du Québec à Montréal, Montréal, Canada; h Département de Psychiatrie, Université de Montréal, Montréal, Canada

**Keywords:** Autism, Cognitive strengths, Structural MRI, Brain maturation, Gyrification, Asperger syndrome

## Abstract

**Background:**

Behavioral, cognitive and functional particularities in autism differ according to autism subgroups and might be associated with domain-specific cognitive strengths. It is unknown whether structural changes support this specialization. We investigated the link between cortical folding, its maturation and cognitive strengths in autism subgroups presenting verbal or visuo-spatial peaks of abilities.

**Methods:**

We measured gyrification, a structural index related to function, in 55 autistic participants with (AS-SOD, *N* = 27) or without (AS-NoSOD, *N* = 28) a speech onset delay (SOD) with similar symptom severity but respectively perceptual and verbal cognitive strengths, and 37 typical adolescents and young adults matched for intelligence and age. We calculated the local Gyrification Index (*l*GI) throughout an occipito-temporal region of interest and independently modeled age and peak of ability effects for each group.

**Results:**

Unique gyrification features in both autistic groups were detected in localized clusters. When comparing the three groups, gyrification was found lower in AS-SOD in a fusiform visual area, whereas it was higher in AS-NoSOD in a temporal language-related region. These particular areas presented age-related gyrification differences reflecting contrasting local maturation pathways in AS. As expected, peaks of ability were found in a verbal subtest for the AS-NoSOD group and in the Block Design IQ subtest for the AS-SOD group.

**Conclusions:**

Irrespective of their direction, regional gyrification differences in visual and language processing areas respectively reflect AS-SOD perceptual and AS-NoSOD language-oriented peaks. Unique regional maturation trajectories in the autistic brain may underline specific cognitive strengths, which are key variables for understanding heterogeneity in autism.

## Highlights

• Subgrouping the autism spectrum (AS) partly accounts for its heterogeneity.
• AS individuals with a speech onset delay (SOD) show perceptual cognitive strengths.
• AS individuals without a SOD show language-related cognitive strengths.
• AS subgroups show unique gyrification patterns in areas related to their strengths.
• Cortical structural maturation may be related to domain-specific strengths in AS.

## 1 Introduction

Autism is a heterogeneous neurodevelopmental condition presenting various cognitive profiles and implicating multiple brain networks. In addition to their challenges, most autistic individuals possess specialized cognitive strengths, from simple peaks of ability to exceptional domain-specific skills (Meilleur et al., 2015). These special abilities mainly encompass two domains of cognition: perception or language. For example, autistic people with a speech onset delay (SOD) perform better in the visual and perceptual subtests (Dawson et al., 2007; Nader et al., 2015; Nader et al., 2016) of typically used intelligence tests (Wechsler, 1991; Wechsler, 1997; Wechsler, 2003; Wechsler, 2008). They also display an enhanced capacity for auditory pure tone discrimination (Bonnel et al., 2010; Jones et al., 2009) and faster processing speed in a visuo-motor inspection time task (Barbeau et al., 2013). Autistic individuals without speech development abnormalities do not show these perceptual abilities, but rather excel in some verbal subtests (Nader et al., 2016; Soulières et al., 2011).

Among the large literature of unique cerebral features in the Autism Spectrum (AS), several studies have revealed enhanced task-related activity in regions not typically underlying the cognitive functions studied (Iuculano et al., 2014; Kana et al., 2006; Samson et al., 2012). For example, functional re-organization of auditory processing in AS depends on speech development history: in response to simple and complex non-social sounds, autistic individuals with a SOD (AS-SOD) show greater primary auditory cortex activity, whereas the AS group without a SOD (AS-NoSOD) preferentially recruits the temporal regions associated with speech and language processing (Samson et al., 2015). These changes in the functional allocation of cerebral resources may aid in cognitive tasks (Iuculano et al., 2014; Soulières et al., 2009). Studies have also reported structural differences associated with the timing of speech onset in AS, mostly concerning regional gray matter volume. AS-SOD individuals show reduced volume in temporal regions associated with language (Lai et al., 2015) and greater gray matter volume in auditory and visual perceptual regions (Hyde et al., 2010; Toal et al., 2010). The same group also shows reduced leftward asymmetry in posterior auditory and language regions paired with elevated rightward asymmetry in the inferior parietal lobule, a region involved in visuospatial processes (Culham et al., 2006). It is therefore plausible that the observed functional and structural differences between AS individuals underlie their perceptual or language-related strengths.

Such findings of structural differences between AS subgroups encourage the search for clues from other structural variables more likely to be related to cognitive function and which may reflect expertise-induced differences. Gyrification refers to the folding of the cortical surface, creating gyri (external structures) and sulci (invaginated surfaces), resulting from the differential growth mechanisms of gray matter layers and the tension constraints imposed on the cortex by axons (Zilles et al., 2013). The link between gyrification and cognitive functioning is exemplified by the positive relationship between high frontal gyrification and good performance in working memory and executive control tasks (Gautam et al., 2015). Regional gyrification in multimodal association regions, such as the prefrontal cortex, positively correlates with global intelligence in two large, distinct samples of typical adults and children (Gregory et al., 2016). High convolution of the cortex has also been related to higher IQ in the frontal lobe, although only in females (Luders et al., 2008). Gyrification may therefore reflect either structurally (genetically) constrained cognitive features or acquired experience-dependent plasticity, which may be specific to the cortical region and the population studied.

Like other structural measures, gyrification changes reflect brain maturation. After a rapid increase of cortical folding around birth and a peak in early childhood (Li et al., 2014; Raznahan et al., 2011), major changes occur throughout adolescence. Raznahan et al. showed a reduction of gyrification at the whole-brain level during this time-period (Raznahan et al., 2011) with more recent studies adding regional precision to this observation. A longitudinal study of typical participants between six and 30 years-old confirmed that most of the cortex undergoes a linear decrease of gyrification (Mutlu et al., 2013), which continues in late adulthood (Hogstrom et al., 2013).

The Gyrification Index (GI), defined as the ratio of the inner to the outer cortical surface, was originally used on single coronal slices (Zilles et al., 1988). This measure initially showed frontal GI differences between AS-SOD and AS-NoSOD (Jou et al., 2010). High-resolution 3D surface-based methods now allow the calculation of a local GI (*l*GI) over the entire cortical surface (Schaer et al., 2008). *l*GI has already been used to assess folding differences between AS and typical children, adolescents (Bos et al., 2015; Libero et al., 2014; Schaer et al., 2013; Schaer et al., 2015; Wallace et al., 2013; Yang et al., 2016), and adults (Ecker et al., 2016; Koolschijn and Geurts, 2016; Libero et al., 2014; Schaer et al., 2015). These studies report mixed results of either lower (Libero et al., 2014; Schaer et al., 2013; Schaer et al., 2015) or higher (Ecker et al., 2016; Wallace et al., 2013) *l*GI in AS across various regions. Inconsistencies may be partly due to the diverse age ranges studied. More recent studies integrate age as a variable of interest and tend to find differences in gyrification maturation between autistic and typical populations (Bos et al., 2015; Libero et al., 2014; Yang et al., 2016).

The goal of the current study was to explore the structural gyrification and maturation features of perception- and language-oriented regions of the brain in two AS subgroups of equivalent intelligence and speech level as adults but presenting a history of speech onset delay (AS-SOD) or a typical speech development (AS-NoSOD). We hypothesized that AS-SOD would have particular structural features associated with their perceptual strengths, whereas AS-NoSOD would have specific features associated with superior language abilities.

## 2 Materials and methods

### 2.1 Participants

Participants from 14 to 30 years of age were recruited from the database of the Center of Excellence for Pervasive Developmental Disorders at the Rivière-des-Prairies Hospital (University of Montreal, Canada), within the framework of three different functional MRI experiments (Barbeau et al., 2015; Samson et al., 2011; Soulières et al., 2009). AS individuals (*N* = 55) met the DSM-IV (American Psychiatric Association, 2000) criteria for autism or Asperger syndrome according to a multidisciplinary evaluation by experienced clinicians (all 55 participants), confirmed by the Autism Diagnostic Interview-Revised (ADI-R, (Lord et al., 1994), eight participants), the Autistic Diagnostic Observation Schedule (ADOS, (Lord et al., 1989), one participant), or both instruments (44 participants). AS individuals were stratified according to the presence or absence of a SOD, irrespective of their diagnosis (i.e. autism or Asperger syndrome). The AS-NoSOD group (*N* = 27) presented normal speech onset, whereas the AS-SOD group (*N* = 28) was characterized by a SOD. Speech acquisition was considered to be delayed when the first spoken words, reported by the main childhood caregiver, occurred after 24 months *or* the first phrases after 33 months, based on the cutoffs used in autistic clinical assessment (ADI-R) and research (Lai et al., 2014; Samson et al., 2015). Absence of SOD was characterized by first words at or before 24 months *and* first phrases at or before 33 months.

Thirty-seven typically developing, non-autistic individuals (TYP) were included in our study. The TYP, AS-SOD, and AS-NoSOD groups were matched for age, Full-Scale IQ, Performance IQ, Verbal IQ, and biological sex (Table 1). The two AS groups did not significantly differ on ADOS or ADI-R subdomain scores (see Table 1 for effect sizes). Since age was a variable of interest in our study, we verified that the age distribution was equivalent between the groups using the Mann-Whitney test, (TYP vs. AS-SOD: *p* = 0.68; TYP vs. AS-NoSOD: *p* = 0.17; AS-SOD vs. AS-NoSOD: *p* = 0.60). Participants using psychoactive medication, presenting neurological or other hereditary psychiatric conditions such as epilepsy, traumatic brain injury and schizophrenia were not included in the study. Additional exclusion criteria for the TYP group included a family history of psychiatric disorders. All participants or parents of minor participants gave written consent and received a compensation for participating in the studies in accordance with Regroupement Neuroimagerie Quebec IRB and the research ethics committee of the Rivière-des-Prairies Hospital approved protocols #2006-0204, #06-07 018, #08-09-003.

**Table 1:** Participants' demographic and socio-communicative characteristics. AS (autism spectrum): autistic individuals with (AS-SOD) and without (AS-noSOD) speech onset delay. TYP: typically developing controls. ADI-R: Autism Diagnostic Interview-Revised. ADOS: autism diagnostic observation schedule. p*: *p*-values of the three-groups ANOVA between TYP, AS-SOD and AS-NoSOD. Effect's sizes of the mean differences between AS-SOD and AS-noSOD are calculated through Cohen's *d*.

Group	TYP	AS-SOD	AS-NoSOD	p*	Cohen's *d*
Sample size (*N* females)	**37** (5)	**28** (3)	**27** (3)		

Age (year)					
Mean (SE)	**20.4** (0.68)	**20.4** (0.78)	**19.8** (0.79)	0.80	0.14
Range	14–28	14–30	14–29		

Full-Scale IQ					
Mean (SE)	**105** (2.2)	**101** (2.5)	**104** (2.6)	0.36	0.23
Range	80–131	66–130	82–129		

Performance IQ					
Mean (SE)	**103** (2.1)	**106** (2.5)	**102** (2.5)	0.10	0.31
Range	79–133	80–131	77–128		

Verbal IQ					
Mean (SE)	**107** (2.4)	**98** (2.7)	**105** (2.8)	0.052	0.43
Range	78–127	72–124	74–134		

ADI-R scores^a^					
Social		22.2	19.9	0.083	0.49
Communication		17.0	15.0	0.086	0.48
Repetitive behavior		6.3	5.7	0.38	0.25

ADOS scores^b^					
Social		10.0	10.3	0.65	0.14
Communication		5.4	5.5	0.83	0.07
Imagination/play		1.1	1.3	0.46	0.23
Repetitive behavior		3.7	3.5	0.63	0.06

a Data missing for four subjects.

b Data missing for 11 subjects.

### 2.2 Cognitive assessment

Participants' IQ was assessed at the time of their enrolment in the database with the Wechsler scales (either Wechsler Adult Intelligence Scale – WAIS-III (Wechsler, 1997) in 43 participants, WAIS-IV (Wechsler, 2008) in 1 participant, or Wechsler Intelligence Scale for Children – WISC-III (Wechsler, 1991) in 42 participants, WISC-IV (Wechsler, 2003) in 6 participants). The difference scores of Verbal IQ subtests (Information, Similarities, Vocabulary, Arithmetic and Comprehension) and Performance IQ subtests (Block Design, Picture Completion, Picture Arrangement, and Digit Symbol-Coding) were included in subsequent analyses. For each subtest, a difference score was computed as the difference score of the subtest minus the mean difference score of other subtests. Each difference score was compared to zero to assess cognitive strengths and weaknesses within the TYP, AS-SOD, and AS-NoSOD groups. Specifically, within-group cognitive strengths were defined here as subtests for which the difference score was significantly higher than zero. The Bonferroni correction for multiple comparisons was used, setting the significance threshold to 0.0056 (0.05 divided by the number of subtests). Finally, between-group differences in cognitive strengths were assessed through Analysis of Variance methods (ANOVAs) and post hoc Tukey test (significance threshold: 0.05).

### 2.3 Imaging parameters

Participants rested comfortably in the scanner (Siemens Trio 3 Tesla, Unité de Neuroimagerie Fonctionnelle, Montréal, Canada) while a high-resolution T1-weighted structural scan was acquired with a MPRAGE sequence (176 slices, 1 mm^3^ voxels). Other sequence parameters slightly differed according to the fMRI experiment that followed the anatomical acquisition: Repetition Time = 2530 ms, Echo Time = 3.5 ms, flip angle = 7° for the experiment from (Barbeau et al., 2015) (*N* = 16 TYP, 14 AS-SOD, 3 AS-NoSOD); Repetition Time = 970 ms, Echo Time = 4 ms, flip angle = 12° for the experiment from (Samson et al., 2011) (*N* = 16 TYP, 15 AS-SOD, 24 AS-NoSOD); Repetition Time = 2530 ms, Echo Time = 3.48 ms, flip angle = 7° for the experiment in (Soulières et al., 2009) (*N* = 9 TYP, 2 AS-SOD, 3 AS-NoSOD).

### 2.4 Surface reconstruction

All data analyses were performed using version 5.3 of FreeSurfer image analysis software (http://surfer.nmr.mgh.harvard.edu/). Anatomical images underwent the first steps of the FreeSurfer surface-based procedure, including removal of non-brain tissue, transformation to standard space, segmentation of gray and white matter structures based on intensity information, and cortical surface reconstruction. Registration to a template enabled anatomical alignment of cortical folds for between-subject comparisons. The pipeline generated two 3-D surfaces per hemisphere in the form of meshes of small triangles: a *pial surface*, i.e. the entire brain surface, and an *outer surface* obtained through pial surface warping that excludes sulcal folds. FreeSurfer registration is very conservative compared to other methods (Dierker et al., 2015), but its accuracy was validated through visual inspection of surfaces to ensure that no artifacts, due to motion over-inclusion of non-brain tissue, or topological defects remained. No participant was excluded at this step.

### 2.5 Local Gyrification Index assessment

Local measurement of gyrification was assessed following the method described by Schaer et al. (2008) and based on the calculation of the GI defined as the ratio of the pial surface to the outer surface. This procedure extends the 2-D definition of the Gyrification Index (Zilles et al., 1988) to a 3-D surface-based index (*l*GI). A spherical region of interest was centered at each vertex (angle point of the triangles forming the mesh) of the outer surface, and the ratio of the pial to the outer surface area enclosed in the sphere was calculated. The sphere radius was set to 25 mm because this length produces particularly contrasted *l*GI distributions and thus allows accurate detection of gyrification anomalies (Schaer et al., 2008). Gyrification normalized brain maps were smoothed using a 10 mm full-width half-maximum Gaussian kernel.

### 2.6 Statistical analysis

**Fig. 1:**
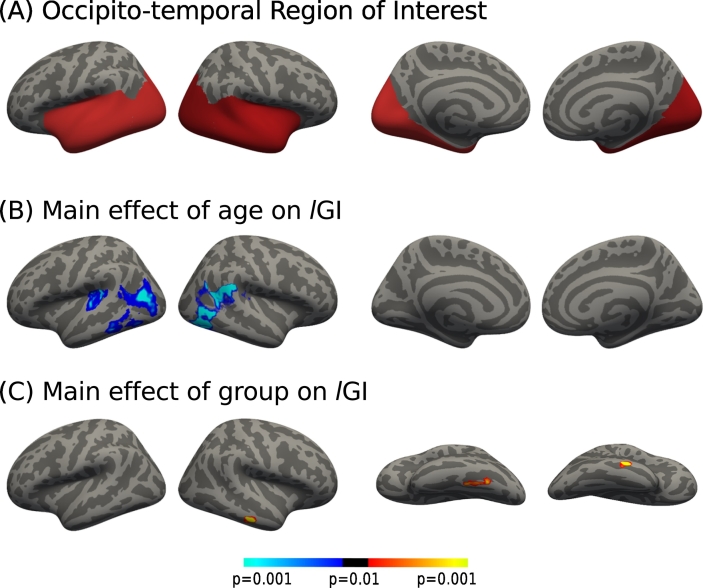
Location of significant age and group effects on gyrification in the occipital and temporal lobes. (A) Occipito-temporal ROI extracted from the PALS-12 brain atlas used to obtain the following results. (B) Main effect of age on gyrification for all participants. Blue clusters indicate a significant inverse correlation between *l*GI and age. (C) Main effect of group on *l*GI. Inflated cortical surface maps (dark gray = sulci; light gray = gyri) represent (from left to right) the left and right lateral and left and right medial views of an average brain map, except in (C) were left and right inferior views are showed. Results are depicted at a significance level of *p* = 0.01 Monte-Carlo corrected for multiple comparisons (cluster-wise *p* = 0.05). (For interpretation of the references to color in this figure legend, the reader is referred to the web version of this article.)

First, we verified that the groups exhibited no differences in terms of total intra-cranial volume (ICV, measured with FreeSurfer), which could have been a confounding factor in the following analysis: mean ICV was respectively 1637, 1671 and 1641 cm^3^ for the TYP, AS-SOD and AS-NoSOD groups (*F*(101,2) = 0.45, *p* = 0.64). Structural brain differences between AS-SOD, AS-NoSOD and TYP groups were expected in regions supporting perceptual and/or language processing. Therefore, subsequent analyses were restricted to a large bilateral Region of Interest (ROI) encompassing the entire temporal (for auditory, secondary visual and language processing) and occipital (for primary visual processing) lobes (Fig. 1A) defined using the PALS-B12 brain atlas (Van Essen, 2005). Group differences in *l*GI were assessed using a General Linear Model (GLM) with the “Different Offset Different Slope” design matrix available in FreeSurfer. This design allowed the independent modeling of the effects of age or any other variable of interest on local gyrification for each group. The F-test computed in the GLM allowed detecting age-by-group interactions, main effect of group and main effect of age on the *l*GI. Clusters showing significant group differences underwent post hoc pairwise *t*-tests to determine the direction of the differences (TYP vs. AS-SOD, TYP vs. AS-NoSOD, AS-SOD vs. AS-NoSOD). These clusters were also used to assess possible localized group differences in the way gyrification evolves with age and a possible relationship between *l*GI and cognitive strengths. Difference-scores of within-group cognitive strengths were used as variables of interest in a similar design as with age. A Monte-Carlo simulation (10,000 iterations, vertex-wise significance level *p* = 0.01, cluster-wise significance level *p* = 0.05, cluster-forming threshold = 15 vertices) was used to correct all results for multiple comparisons. The mean *l*GI of clusters showing differences in the age-by-group interaction was extracted for each subject and plotted against age to allow better visualization of gyrification changes with age for each group. Linear regression was calculated using the least squares method and its significance was assessed with an F-test at a 0.05 significance level. A preliminary analysis was performed to ensure that parameters that differed between the three fMRI experiments did not have a significant effect on *l*GI measures. The factor ‘dataset of origin’ (stating which of the three fMRI experiments each subject participated in) was entered into the GLM of FreeSurfer and the analysis revealed no main effect of experiment on gyrification even at an uncorrected level of *p* = 0.01.

## 3 Results

### 3.1 Gyrification differences in AS-SOD and AS-NoSOD in occipital and temporal lobes

The following results were obtained using an occipito-temporal ROI (Fig. 1A). First, the main effect of age on gyrification was investigated. We observed predicted age-dependent *l*GI reductions, which here encompassed various areas of the superior and inferior temporal and middle and inferior lobes on the left. On the right, one large cluster which extended from the posterior cingulate gyrus and the cuneus to the lingual and fusiform gyri was found (Fig. 1B and Supplementary Table A).

**Fig. 2:**
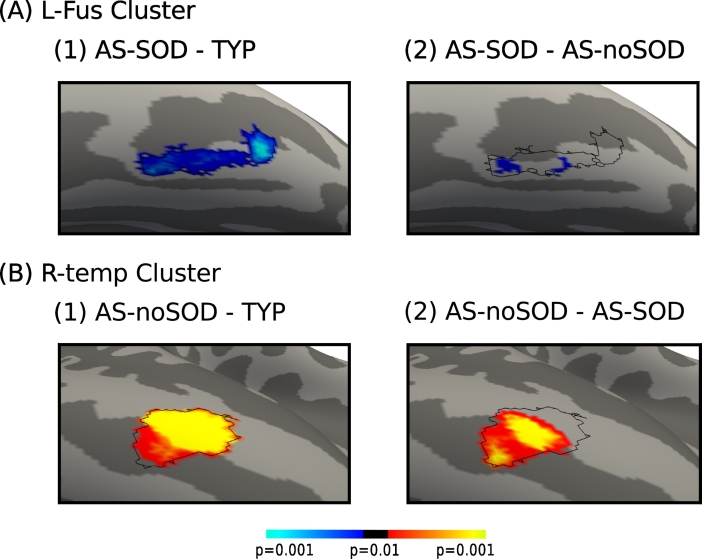
Differences in gyrification between AS-SOD, AS-NoSOD, and TYP groups. Only significant differences in *l*GI between groups are shown. Left fusiform (L-fus) cluster: AS-SOD vs. TYP (A1) and AS-SOD vs. AS-NoSOD (A2). Blue colors indicate lower gyrification in the AS-SOD group. Right middle-temporal (R-temp) cluster: AS-NoSOD vs. TYP (B1) and AS-NoSOD vs. AS-SOD (B2). Red colors indicate higher gyrification in AS-NoSOD. Magnifications of left and right inflated cortical surface maps (dark gray = sulci; light gray = gyri). The black solid lines indicate the limits of L-fus and R-temp clusters as defined in Fig. 1. Results are depicted at a significance level of *p* = 0.01 Monte-Carlo corrected for multiple comparisons (cluster-wise *p* = 0.05). AS: autism spectrum. SOD: speech onset delay. TYP: typical individuals. (For interpretation of the references to color in this figure legend, the reader is referred to the web version of this article.)

A main effect of group on *l*GI was found only in two localized clusters, the first in the left fusiform area (L-fus cluster) and the second in the right anterior part of the middle temporal gyrus (R-temp cluster, Fig. 1C and Table 1). Post hoc analyses revealed that the *l*GI was significantly reduced in the AS-SOD group in the whole L-fus cluster surface when compared to the TYP group, and in small patches of the same cluster when compared to the AS-NoSOD group (Fig. 2A and Table 2). Most of the R-temp cluster surface showed higher gyrification in the AS-NoSOD group compared to both TYP and AS-SOD groups (Fig. 2B and Table 2).

**Table 2:** Clusters of significant group differences in gyrification in the occipito-parietal lobes. Reported are (from left to right) the cluster abbreviation as used in the main text if applicable, cluster area size in mm^2^ and in vertices, the maximum vertex and its MNI (Montreal Neurological Institute) coordinates, the Cluster-Wise *P*-value (CWP) and the cluster location (hemisphere and FreeSurfer given anatomical region).

Abbreviation	Size (mm^2^)	Number of vertices	Vertex Max	Peak MNI coordinates			CWP	Hemisphere	Anatomical region
				X	Y	Z			
F-test on *l*GI									
Main effect of age	4169	8097	44,644	60	−38	23	0.0004	R	Supramarginal
	1716	3129	141,942	−41	−68	12	0.0002	L	Inferior-parietal
	972	2476	141,743	−42	−35	18	0.0002	L	Supramarginal
	560	865	114,273	−56	−41	−17	0.0002	L	Inferior-temporal
	266	469	118,300	−42	−60	−5	0.012	L	Inferior-temporal
Main effect of group									
**R-temp**	**225**	**366**	**17,622**	**61**	**−21**	**−20**	**0.03**	**R**	**Middle-temporal**
**L-fus**	**219**	**363**	**90,732**	**−43**	**−59**	**−17**	**0.04**	**L**	**Fusiform**
Age-by-group interaction									
No significant cluster									

Post hoc t-tests on *l*GI									
TYP > AS-SOD									
	219	363	40,391	−42	−16	−16	0.0001	L	Fusiform
AS-SOD > TYP									
No significant cluster									
TYP > AS-NoSOD									
No significant cluster									
AS-NoSOD > TYP									
	213	345	17,622	61	−21	−20	0.0001	R	Middle-temporal
AS-SOD > AS-NoSOD									
No significant cluster									
AS-NoSOD > AS-SOD									
	146	234	1799	60	−22	−20	0.0001	R	Middle-temporal
	18	28	128,008	−40	−42	−21	0.0001	L	Fusiform
	8	15	90,788	−39	−51	−20	0.001	L	Fusiform

No region showed a significant age-by-group interaction at the level of this large occipito-temporal ROI (Table 1).

### 3.2 Developmental trajectories of gyrification

**Fig. 3:**
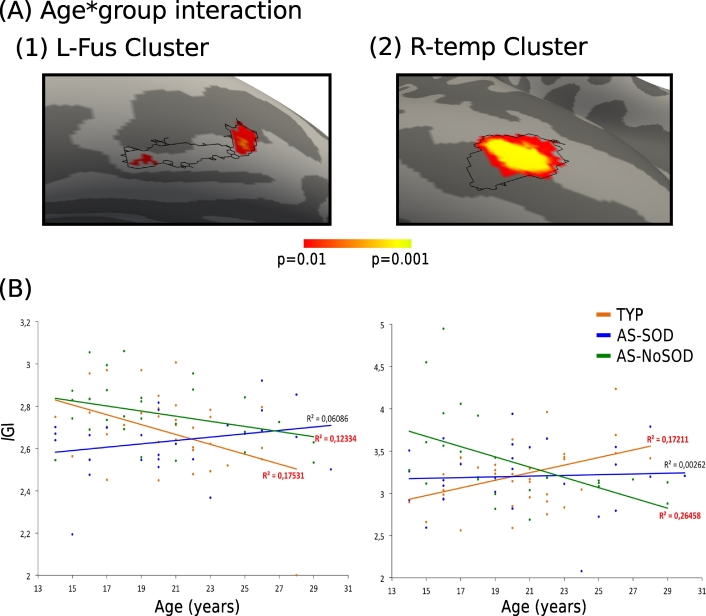
Differences in the maturation of gyrification in left-fusiform and right-temporal clusters. (A) Significant age-by-group interactions inside the left fusiform (L-fus, A1) and right middle-temporal (R-temp) clusters. Magnifications of left and right inflated cortical surface maps (dark gray = sulci; light gray = gyri). The black solid lines indicate the limits of L-fus and R-temp clusters as defined in Fig. 1. (B) Corresponding scatter plots of the correlations between age and *l*GI for L-fus (left) and R-temp (right) sub-areas showing an interaction. Individual mean *l*GI are plotted against age. Linear regressions are depicted by solid lines. The correlation coefficient R^2^ shown next to each linear regression curve is depicted in red when the associated *p*-value was lower than 0.05. AS: autism spectrum. SOD: speech onset delay. TYP: typical individuals.

In order to explore what underlies group differences in *l*GI in the middle temporal (R-temp) and fusiform (L-fus) gyri clusters, we took a closer look at local gyrification maturation patterns during adolescence and young adulthood in AS and TYP groups. A GLM analysis using age as a variable of interest was run on the L-fus and R-temp clusters. This revealed a significant age-by-group interaction in a major part of the R-temp cluster, driven by a difference between the AS-NoSOD group and the two other groups (*p* < 0.001) and in a smaller surface of the L-fus cluster, driven by a difference between the AS-SOD and TYP groups (*p* < 0.001, Fig. 3A). To visualize the maturation patterns of these surfaces, we plotted their mean *l*GI against age for each group (Fig. 3B). Sub-clusters in the L-fus surface showed a noticeable decrease in gyrification in the control (R^2^ = 0.18, *p* < 0.01) and in the AS-NoSOD (R^2^ = 0.12, *p* = 0.05) groups, whereas no change with age was detected in the AS-SOD group. For the R-temp cluster, the *l*GI significantly increased with age in the TYP group (R^2^ = 0.17, *p* = 0.01), and in contrast decreased in the AS-NoSOD group (R^2^ = 0.26, *p* < 0.01). The AS-SOD group showed an intermediate pattern with no significant age-related changes, and did not significantly differed from the TYP group.

### 3.3 Relationship between AS group-specific cognitive strengths and gyrification

To assess whether gyrification features could be linked to the specific cognitive strengths exhibited by AS-SOD and AS-NoSOD subgroups according to the literature (respectively in the Block Design subtest and language-related subtests of the Wechsler IQ scale), we first investigated whether the cognitive profiles of our experimental groups differed (Table 3).

**Table 3:** Cognitive profiles of the TYP, AS-SOD and AS-NoSOD groups. Peak value is the group mean of the difference between the individual subtests scores and the mean of all other IQ subtests available for each participant. Values in bold represent significant group cognitive strengths. SD: standard deviation. p*: when significant, *p*-values of the *t*-test between peak value and a normal distribution around zero. Effect's sizes are calculated through Cohen's *d*. Pic.: picture.

Group	TYP				AS-SOD				AS-NoSOD			
	Peak value	SD	p*	*d*	Peak value	SD	p*	*d*	Peak value	SD	p*	*d*
Verbal subtests												
Similarities	0.40	1.92	–		0.49	2.14	–		1.36	2.37	–	
Vocabulary	1.05	2.17	–		−0.39	2.71	–		0.45	3.14	–	
Arithmetic	0.26	2.23	–		0.68	3.21	–		0.83	3.33	–	
Information	0.44	2.18	–		0.21	3.33	–		**2.68**	**3.03**	**1.9**^**E−4**^	**0.88**
Comprehension	−0.15	2.05	–		−3.47	3.12	4^E−6^	1.11	−0.98	2.44	–	

Performance subtests												
Coding	−1.82	3.21	0.002	0.57	−2.33	4.23	–		−3.47	3.13	4^E−6^	1.11
Block design	0.55	2.43	–		**3.88**	**3.11**	**4.4**^**E−7**^	1.25	1.36	3.38	–	
Pic. completion	−0.47	2.54	–		0.44	2.99	–		−0.99	2.08	–	
Pic. arrangement	0.26	2.16	–		0.58	4.04	–		1.22	4.01	–	

**Fig. 4:**
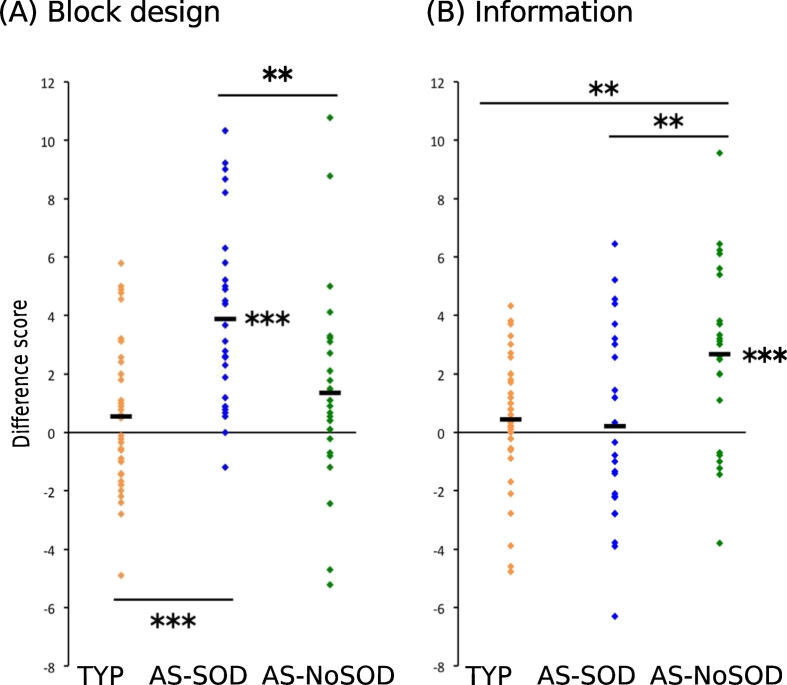
Cognitive strengths of AS subgroups. Individual difference-scores for the Block design (A) and Information (B) subtests of the Wechsler IQ scales. The difference score is defined as the difference between the score of a particular subtest and the mean score of all the other subtests. Each square represents a single subject from the TYP (orange), AS-SOD (blue) and AS-NoSOD (green) groups. Each group mean is represented by a black line. Significant differences between the difference-scores and zero (*t*-test) and between groups (post-ANOVA Tukey test) are highlighted with stars. **: *p* ≤ 0.01; ***: *p* ≤ 0.001. AS: autism spectrum. SOD: speech onset delay. TYP: typical individuals. (For interpretation of the references to color in this figure legend, the reader is referred to the web version of this article.)

The AS-SOD group had a significantly higher score on the Block Design subtest than on other subtests (Block difference-score = 3.88, *t*(27,1) = 6.6, *p* < 0.001). The AS-NoSOD group demonstrated one verbal cognitive strength (Information difference-score = 3.0, *t*(24,1) = 4.42, *p* < 0.001). Finally, the TYP group did not show any particular cognitive strength. Analysis of Variance showed that cognitive strengths in autism were group-specific, because they differed between the three groups (Fig. 4). Specifically, the Block Design difference-score (*F*(2,91) = 10.2; *p* < 0.001) was significantly higher in AS-SOD compared to the AS-NoSOD (*p* = 0.01) and TYP (*p* < 0.001) groups (Fig. 4A). For the Information subtest (*F*(2,84) = 6.1; *p* = 0.003), significantly higher performance in AS-NoSOD compared to AS-SOD (*p* = 0.008) and TYP (p = 0.008) groups was observed (Fig. 4B).

Finally, we explored group-by-difference-score interaction on *l*GI values for the AS-SOD (Block Design) and AS-NoSOD (Information) cognitive strengths, by re-running the FreeSurfer analysis pipeline across L-fus and R-temp clusters using difference-scores as variables of interest. No group-by-difference score interaction was foundrst investigated whether the cognitive profiles oftests (Table 3).

## 4 Discussion

We explored the unique structural features of the autistic brain focusing on gyrification, its maturation and its link with the specific cognitive strengths of AS subgroups throughout adolescence and early adulthood. We argue that atypical maturation trajectories in the autistic brain could be related to the cognitive strengths specific to AS subgroups, which may in part account for the high heterogeneity of the spectrum.

### 4.1 Gyrification changes as an index of brain maturation

In the general population, the timing and speed of cortical unfolding during adolescence differs according to brain region (Alemán-Gómez et al., 2013; Klein et al., 2014; Su et al., 2013). The most substantial decreases have been described in the frontal lobe (Alemán-Gómez et al., 2013; Klein et al., 2014), the occipital cortex (Alemán-Gómez et al., 2013), and in precentral and temporal regions (Klein et al., 2014). The current study was no exception, as decreases in gyrification with age were detected, even if frontal and precentral areas were not included in the analysis. The locations of the decreases found in the occipital and temporal lobes confirmed previous findings in the typical and AS populations (Wallace et al., 2013). In addition, within focal brain regions where a main effect of group on gyrification was found, post hoc analyses revealed significant age-by-group interactions, uncovering unique gyrification maturation patterns in each AS group. This is in agreement with previous studies that examined the effect of age on gyrification in autism (Bos et al., 2015; Libero et al., 2014; Yang et al., 2016) and suggest that the main focus of structural studies in AS should be differential brain maturation pathways rather than mere regional differences. In the frontal cortex of AS individuals, an atypical increase in gyrification between four and 12 years of age (Yang et al., 2016) precedes an abnormal decrease related to atypical connectivity in adolescents from eight to 18 years old (Bos et al., 2015). In the current study, clusters situated in more infero-posterior regions demonstrated atypical maturation patterns in older AS-SOD and AS-NoSOD participants. This constitutes an additional argument in favor of the consideration of developmental trajectories in AS structural studies, which might differ from typical pathways from pre-natal to late adolescence development, and even in aging (Koolschijn and Geurts, 2016).

### 4.2 Regional *l*GI differences in AS are associated with respective cognitive strengths

We observed small areas of gyrification differences between the TYP and each AS group, in agreement with recent studies suggesting overall similarity between AS and TYP structural cortices (Koolschijn and Geurts, 2016), despite subtle differences (Dierker et al., 2015; Ecker et al., 2016; Libero et al., 2014; Via et al., 2011). Using brain volume, a meta-analysis (Yu et al., 2011) and various experimental studies (Floris et al., 2016; Hyde et al., 2010; Lai et al., 2014; Toal et al., 2010) also identified distinct patterns of structural differences when comparing AS-SOD and AS-NoSOD to controls. Thus, even if building subgroups based on SOD obviously reduces statistical power, it also increases the specificity of results. This power pitfall was addressed here by restricting the analysis to a large occipito-temporal ROI as structural differences were expected in visual and/or auditory areas for AS-SOD and in language regions for AS-NoSOD. Our results indeed suggest an association between anatomical changes and cognitive strengths in autism.

AS-SOD showed a peak of ability in the visual Block Design task, confirming previous reports (Caron et al., 2006; Dawson et al., 2007; Ehlers et al., 1997; Meilleur et al., 2014; Nader et al., 2015). This peak reflects the general perceptual abilities of the AS-SOD group, like superior visual search capacity (Joseph et al., 2009; Kemner et al., 2008), as well as better performance on visual discrimination tasks, than non-autistics (Bertone et al., 2005). They also show enhanced activity and functional connectivity in visual expertise regions according to studies using the Raven Progressive Matrices test (Simard et al., 2015; Soulières et al., 2009) and to a meta-analysis of 26 neuroimaging studies of visual processing (Samson et al., 2012). The area showing specific gyrification aspects in AS-SOD compared to TYP, and to a lower extent to AS-NoSOD, is implicated in the integration and manipulation of visual features (Wandell et al., 2007), and in the recognition of visually presented objects (Op de Beeck et al., 2008). Within the infero-temporal cortex, the fusiform is particularly important for the development of perceptual expertise (Gauthier et al., 1999).

Alternatively, the right middle temporal region, displaying *l*GI differences between AS-NoSOD and TYP in the current study, belongs to the speech processing network and specifically responds to auditory words and syllables (Binder et al., 1991; Liebenthal et al., 2000). These region is part of the so-called ‘temporal voice area’ (Belin et al., 2000; Pernet et al., 2015) showing preferential responses to vocal sounds (Belin et al., 2002; Samson et al., 2010). Behaviorally, AS-NoSOD individuals demonstrated heightened abilities in a language-related subtest of the IQ scale, confirming previous findings of verbal strengths (Chiang et al., 2014; Ehlers et al., 1997; Nader et al., 2016). In a study using a reasoning task, AS-NoSOD preferentially used a semantic rather than visuo-spatial strategy (Sahyoun et al., 2009). They also showed more activity than AS-SOD or controls in regions associated with language processing when listening to non-social speech-like stimuli (Samson et al., 2015). The differences in *l*GI within the temporal cluster are therefore consistent with the interest and abilities in language-related tasks demonstrated by AS-NoSOD individuals.

### 4.3 Domain-specific strengths in AS are linked to cortical development

Among non-autistic individuals, some studies may indicate a link between *l*GI variations with time and experience-dependent plasticity. Specific gyrification patterns have been found in experienced divers (Zhang et al., 2016) and meditation experts (Luders et al., 2012), together with a correlation between *l*GI and years of intensive practice in regions specific to expertise. However, the direction of *l*GI changes and their correlation with experience were not consistent between studies. Even if no direct relationship was found between within-group cognitive peaks and areas of specific gyrification features, their locations suggest that gyrification and domain-general strengths may be somehow related in autism. It is possible that regions of *l*GI particularities underlie perceptual and language functions different from those assessed by the Wechsler IQ tests. The specific age-related differences described here between AS subgroups and controls in regions associated with their respective cognitive, behavioral, and functional superiorities suggest that the evolution of gyrification may reflect the development of specific expertise in AS too. Documented age-related changes in the functional connections originating from the fusiform face area parallel our structural results: connectivity strength between this seed and other visual regions decrease from adolescence to early adulthood in TYP, whereas it increases in AS (Lynn et al., 2018). This is not surprising as gyrification had been associated with structural connectivity strength (Zilles et al., 2013). Altogether, these results reinforce the claim that gyrification maturation is linked to abilities development in AS, through connectivity plasticity.

However, neither previous nor current results allow unequivocal conclusions concerning the meaning of the direction of *l*GI changes with age. More accurate modeling, on larger age ranges, may partly address this issue. For example, although it is convenient to compare clinical groups, linear regression may not provide the best fit for maturation curves (Klein et al., 2014). Finally, longitudinal studies of autistic children and adolescents, with and without a SOD, and in light of various structural metrics, may provide further insight into the unique features of brain maturation across regions in autism.

## 5 Conclusion

We found unique gyrification features in cerebral regions associated with the specific domains of cognitive strengths of AS-SOD and AS-NoSOD groups. Differentiating AS individuals according to their speech acquisition history, which was the former basis of the DSM-IV diagnostic categories of prototypical autism and Asperger's syndrome (American Psychiatric Association, 2000), remains relevant. Indeed, symptomatic and structural heterogeneity in autism may partially arise from increased experience-dependent neural plasticity targeting either perceptual or speech cortical regions (Mottron et al., 2014). Structural brain metrics, such as gyrification and their evolution throughout life may support this hypothesis.

The following is the supplementary data related to this article.

Supplementary Table A Clusters showing significant age-related decreases in gyrification for all the participants. TYP, AS-SOD and AS-NoSOD groups are pooled in one group here. Reported are (from left to right) cluster number (#), cluster area size in mm^2^ and in vertices, the maximum vertex and its MNI (Montreal Neurological Institute) coordinates, the Cluster-Wise *P*-value (CWP) and the cluster location (hemisphere and FreeSurfer given anatomical region). Supplementary Table A
