# Supplementary material for: Gyrification changes are related to cognitive strengths in autism
Source: Neuroimage Clin. 2018 Aug 4;20:415–23. doi: 10.1016/j.nicl.2018.04.036 (PMC6095946; doi:10.1016/j.nicl.2018.04.036)
Supplement: Supplementary Table A — Clusters showing significant age-related decreases in gyrification for all the participants. TYP, AS-SOD and AS-NoSOD groups are pooled in one group here. Reported are (from left to right) cluster number (#), cluster area size in mm2 and in vertices, the maximum vertex and its MNI (Montreal Neurological Institute) coordinates, the Cluster-Wise P-value (CWP) and the cluster location (hemisphere and FreeSurfer given anatomical region). [file mmc1.docx]

| # | **Size (mm^2^)** | **Number of vertices** | **Vertex Max** | **Peak MNI coordinates** | | | **CWP** | **Hemis-phere** | **Anatomical region** |
| --- | --- | --- | --- | --- | --- | --- | --- | --- | --- |
|  |  |  |  | X | Y | Z |  |  |  |
| Main effect of age on *l*GI | | | | | | | | | |
| 1 | 21648 | 47103 | 11647 | -48 | -4 | 47 | 0.0002 | L | Precentral |
| 2 | 1649 | 4038 | 141743 | -42 | -35 | 18 | 0.0002 | L | Supramarginal |
| 3 | 300 | 490 | 122712 | -61 | -41 | -11 | 0.03 | L | Middle temporal |
| 4 | 16281 | 34930 | 43119 | 37 | 5 | 37 | 0.0002 | R | Caudal middle frontal |
| 5 | 1490 | 3267 | 36715 | 19 | -42 | 62 | 0.0002 | R | Superior parietal |
| 6 | 1164 | 1993 | 70934 | 41 | 33 | 20 | 0.0002 | R | Rostral middle frontal |
| 7 | 846 | 1451 | 80366 | 11 | 43 | 43 | 0.0002 | R | Superior frontal |
| 8 | 323 | 860 | 85504 | 7 | -47 | 43 | 0.02 | R | Precuneus |
| 9 | 320 | 817 | 80828 | 5 | -30 | 64 | 0.02 | R | Paracentral |

**Supplementary Table A:** **Clusters showing significant age-related decreases in gyrification for all the participants.** TYP, AS-SOD and AS-noSOD groups are pooled in one group here. Reported are (from left to right) cluster number (#), cluster area size in mm^2^ and in vertices, the maximum vertex and its MNI (Montreal Neurological Institute) coordinates, the Cluster-Wise P-value (CWP) and the cluster location (hemisphere and FreeSurfer given anatomical region).
